# Supplementary material for: Different patterns of clonal evolution among different sarcoma subtypes followed for up to 25 years
Source: Nat Commun. 2018 Sep 10;9:3662. doi: 10.1038/s41467-018-06098-0 (PMC6131146; doi:10.1038/s41467-018-06098-0)
Supplement: Supplementary file 1 — Supplementary Information [file 41467_2018_6098_MOESM1_ESM.pdf]

Supplementary Information

Different patterns of clonal evolution among different sarcoma subtypes followed for up to 25 years

Hofvander et al.

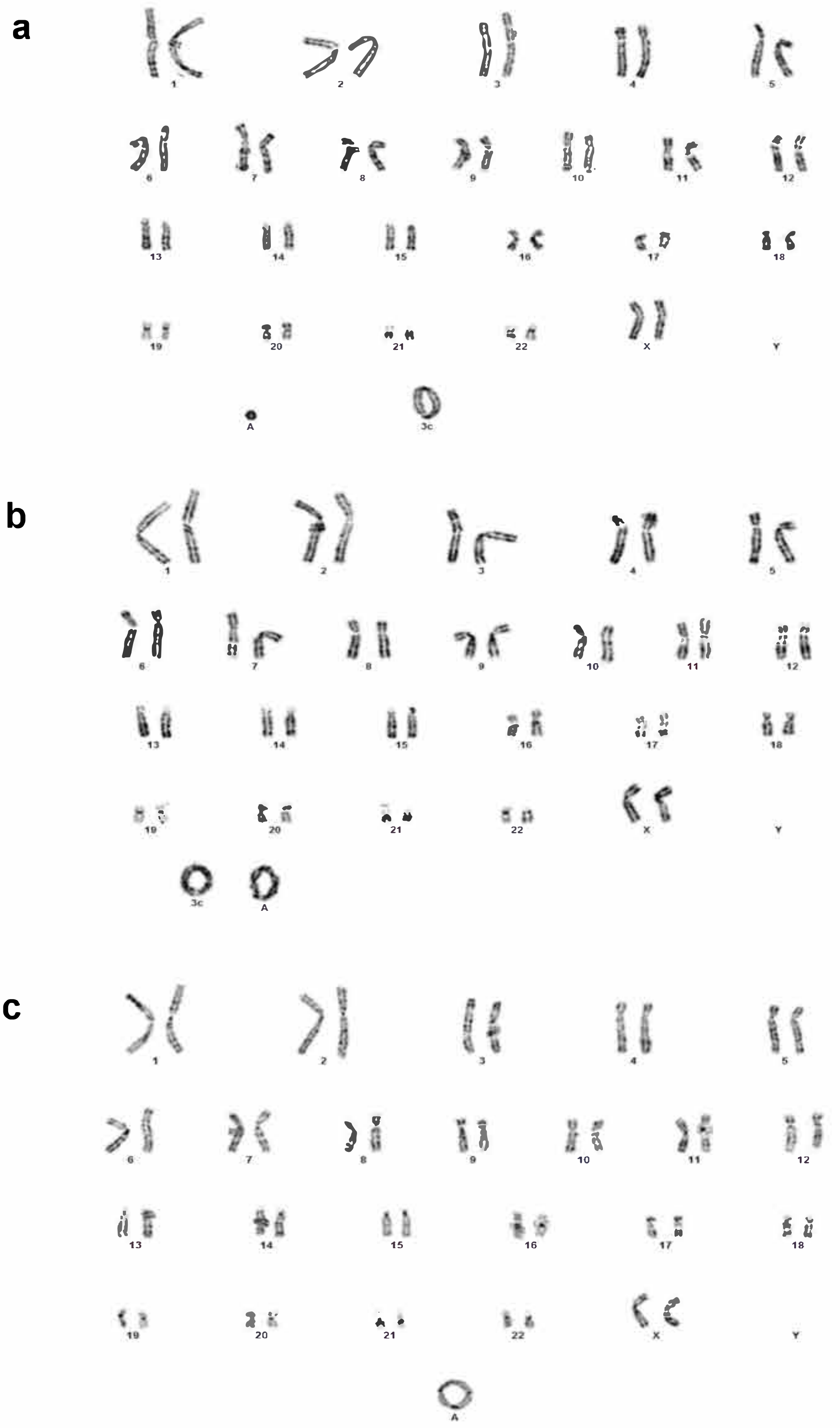

Supplementary Fig. 1. Single cell variation.
Variation at the single cell level in a well-differentiated liposarcoma (local recurrence 2 of Case 10), as demonstrated by G-banding analysis. (a) Cell with one large and one small ring chromosome; (b) Cell with two large ring chromosomes; (c) Cell with only one ring chromosome.

a

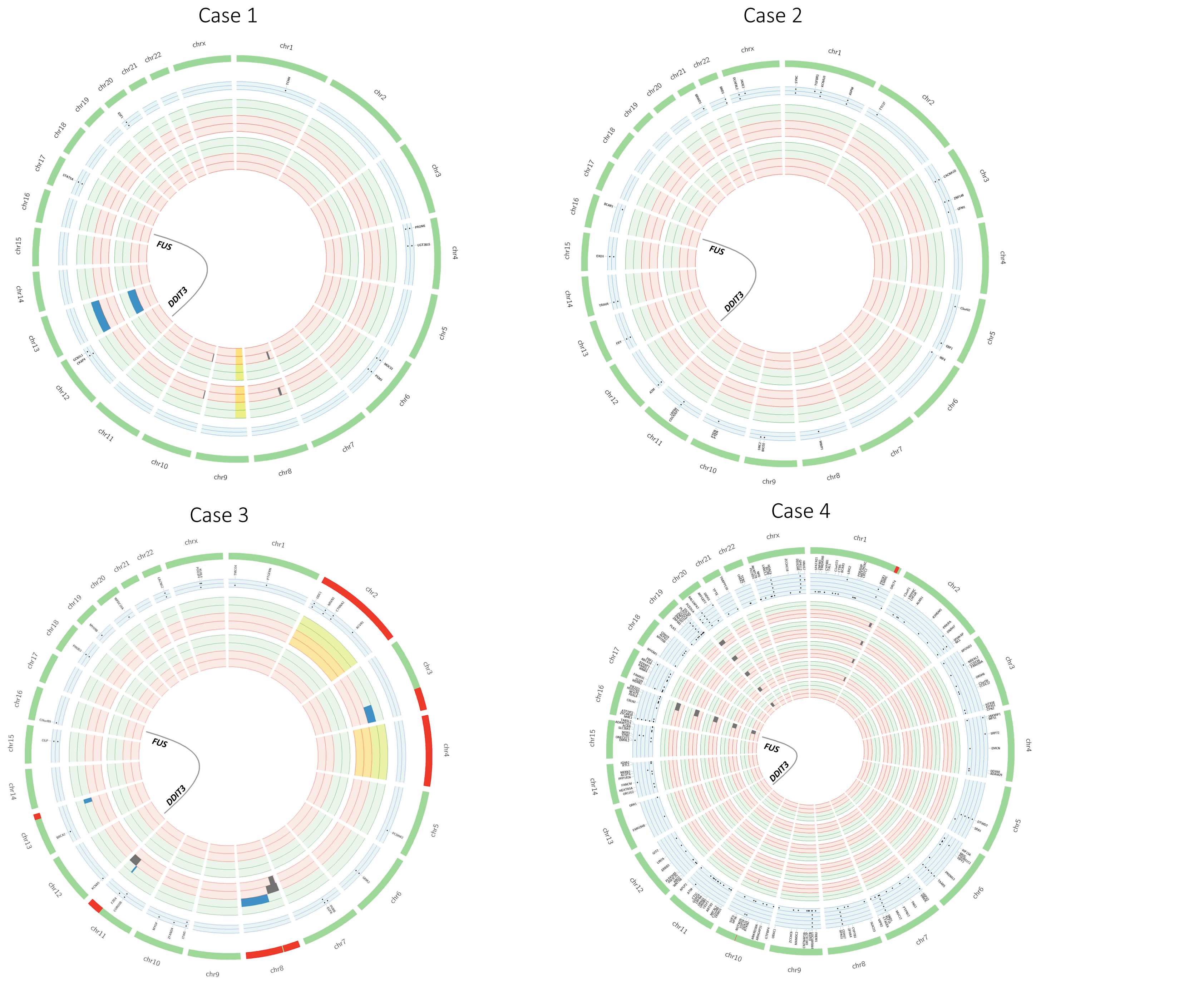
Case 1
Case 2
Case 3
Case 4
chr1
chr2
chr3
chr4
chr5
chr6
chr7
chr8
chr9
chr10
chr11
chr12
chr13
chr14
chr15
chr16
chr17
chr18
chr19
chr20
chr21
chr22
chrx
FUS
DDIT3

b

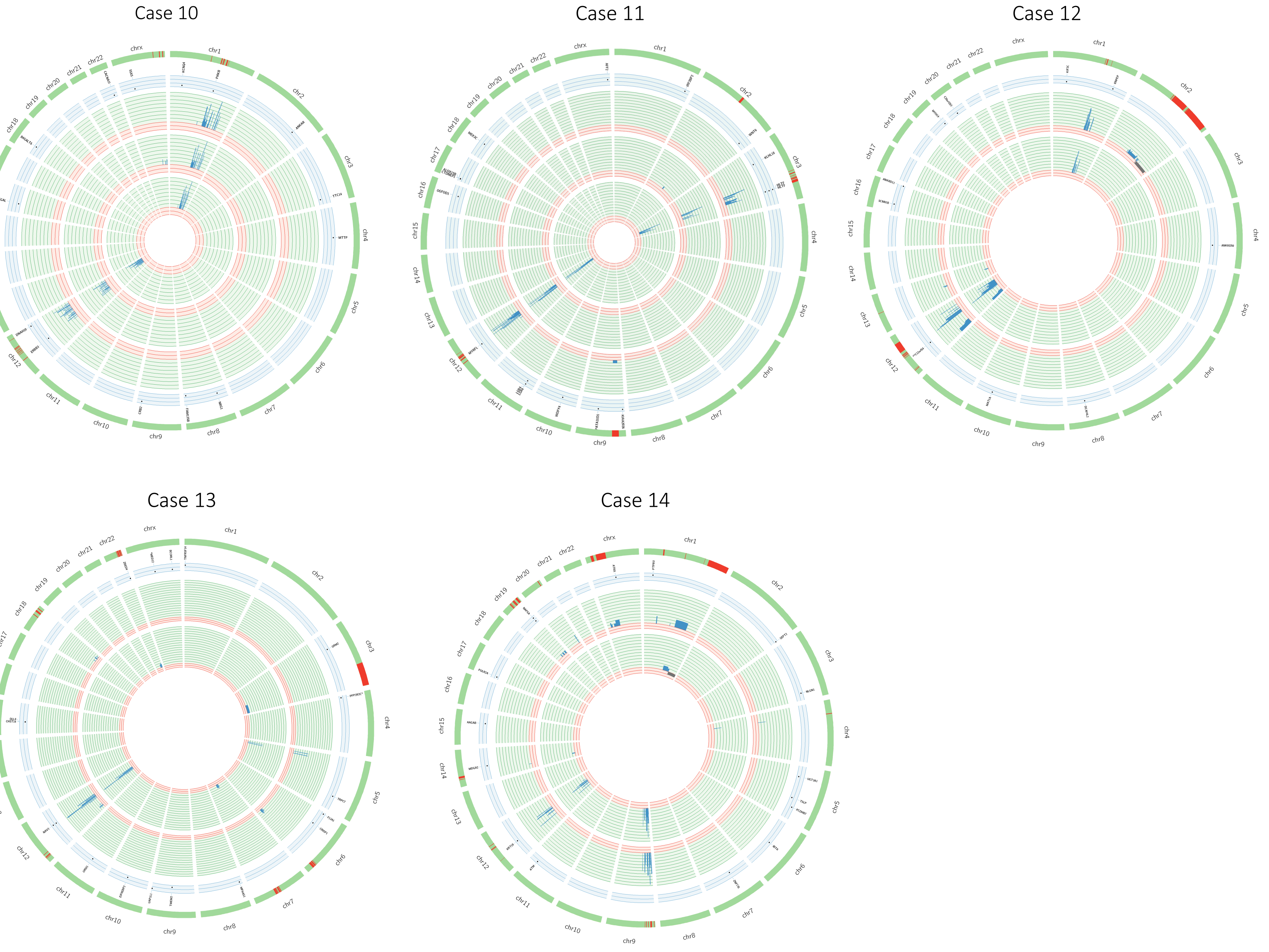

c

Case 15

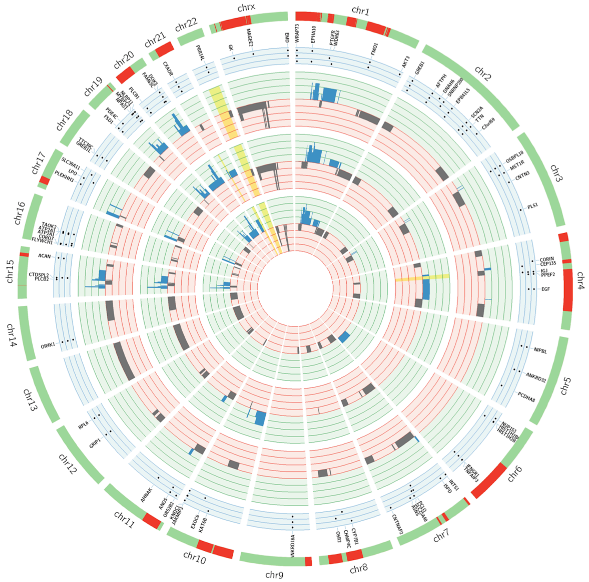

Case 16

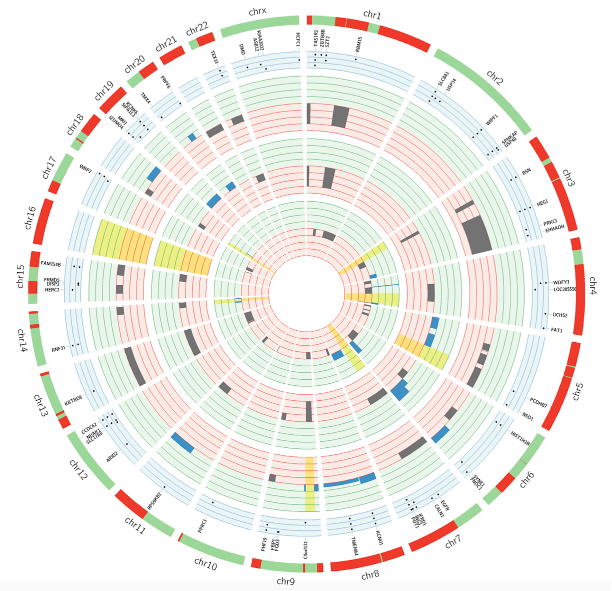

Case 17

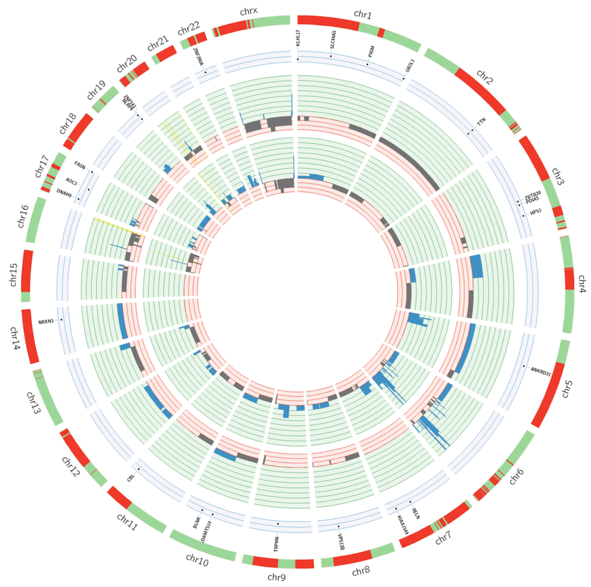

Case 18

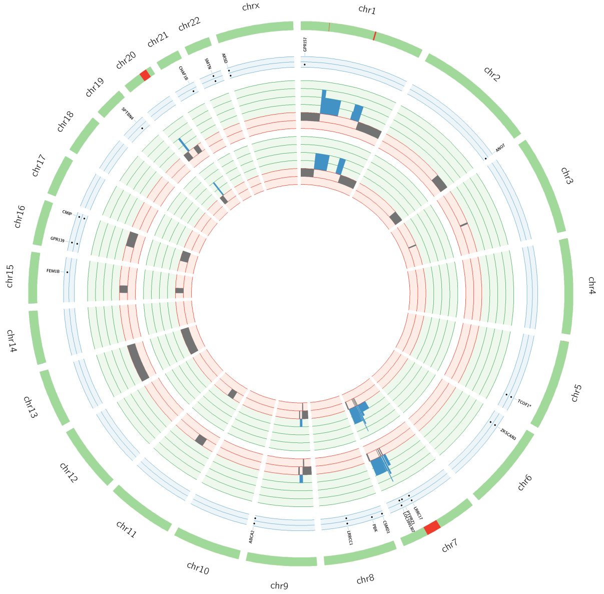

Case 19

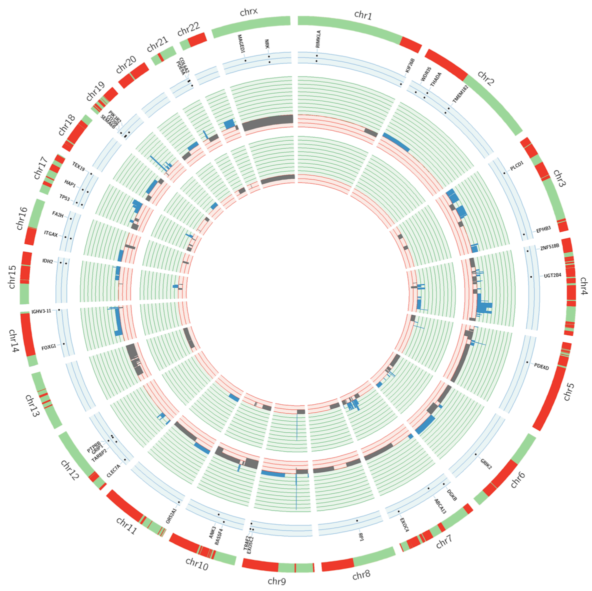

Case 20

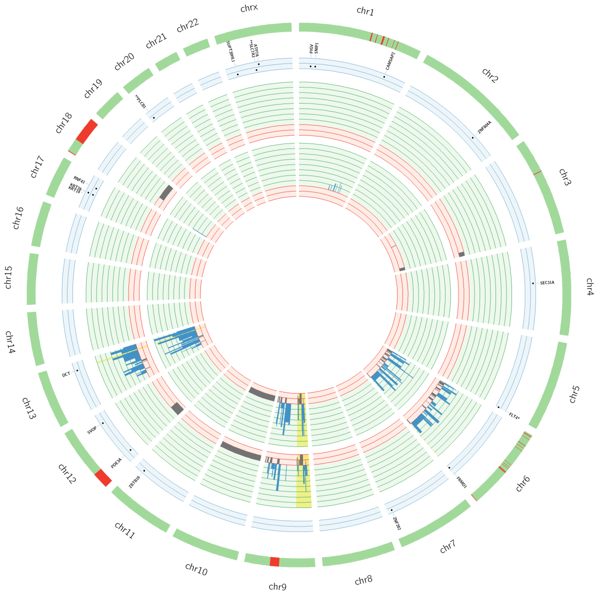

Supplementary Fig. 2. Circos plots summarizing SNP array and whole exome sequencing results.
The red/green inner circles represent the location and amplitude of the genomic changes at SNP array (GCS); blue is gain, grey is loss and yellow background indicates LOH. The circles are ordered chronologically, starting from the center with the first lesion. The light blue circles represent the location of the variants reported by the WES in the same order. Red on the green schematic chromosomes represents differences in GCS between lesions. (a) Fusion-driven sarcomas; (b) amplicon-driven sarcomas; (c) sarcomas with complex genomes.

Supplementary Table 1. Single cell variation in myxoid and well-differentiated liposarcomas, assessed by chromosome banding

Case No.	Sample[a]	Chromosome number in stemline and sidelines[b]	No. of Ac[c]	No of cells with NCSA[d]	No. of chromosomes in AC[c] <45	45	46	47	48	49	>49
Myxoid liposarcoma											
1A	PT	46	11	0			11				
1B	LR2	46	11	0			11				
2A	PT	46	25	0			25				
2B	Met1	46	25	0		2	23				
2C	Met2	46	10	0			10				
2D	Met3	46	4	1		1	3				
5A1	PT	46	6	0			6				
5B	LR1	46	17	3	1		16				
7A1	PT	46	75	0			75				
7A2	PT	46	25	0			25				
7A3	PT	46	24	0			24				
8A	PT	47	23	0				23			
8B	Met1	47	25	0				25			
9A	PT	46	17	0			17				
9B	Met1	46	19	0			19				
**Total**			**317**	**4**	1	3	265	48	0	0	0
Well-differentiated liposarcoma											
10A1	PT	47-49/47/46	20	9			2	4	8	6	
10B	LR1	49	1	0						1	
10C	LR2	47-48	15	1		1		8	6		
11B	LR1	47	8	2				5	1		2
11C	LR2	45-50/88-94	17	14		1	2	4	5	2	3
11D	LR3	46-49/45-46	14	0		1	7	4	2		
12A1	PT	43-49/44-45/84-89	20	20	2	2				1	15
12B	LR1	76-88	11	3	1		1		1		8
13A1	PT	47/48-49/49-52/51	20	9				2	3	4	11
13B	LR2	45-47	22	2		2		12	4	1	3
14A1	PT	47-48/80-89	10	6	1		1	1	4		3
14B	LR1	48-50	9	4				1	4	3	1
**Total**			**167**	**70**	4	7	13	41	38	18	46

[a] PT = primary tumor; LR = local recurrence

[b] Only the modal chromosome number in each clone is shown here. Clones are separated by /. For full karyotypes, see Supplementary Table 6.

[c] AC = abnormal cells

[d] NCSA = non-clonal structural aberrations

Supplementary Table 2: Jaccard Index

WDLS

Sample	10A	10B	10C	11B	11C	11D	12A	12B	13A	13B	14A	14B
10A	1,00											
10B	0,69	1,00										
10C	0,94	0,78	1,00									
11B	0,07	0,08	0,07	1,00								
11C	0,06	0,07	0,07	0,57	1,00							
11D	0,05	0,07	0,06	0,67	0,39	1,00						
12A	0,19	0,17	0,20	0,23	0,18	0,23	1,00					
12B	0,08	0,07	0,08	0,05	0,03	0,05	0,25	1,00				
13A	0,02	0,02	0,02	0,15	0,07	0,13	0,05	0,02	1,00			
13B	0,03	0,04	0,04	0,08	0,08	0,06	0,10	0,04	0,11	1,00		
14A	0,17	0,15	0,17	0,03	0,02	0,04	0,09	0,06	0,01	0,02	1,00	
14B	0,16	0,14	0,16	0,02	0,01	0,04	0,08	0,06	0,02	0,02	0,40	1,00

WDLS: Chr12

Sample	10A	10B	10C	11B	11C	11D	12A	12B	13A	13B	14A	14B
10A	1,00											
10B	0,72	1,00										
10C	0,97	0,72	1,00									
11B	0,15	0,19	0,16	1,00								
11C	0,12	0,15	0,14	0,70	1,00							
11D	0,15	0,19	0,17	0,99	0,69	1,00						
12A	0,16	0,14	0,17	0,29	0,22	0,28	1,00					
12B	0,12	0,11	0,13	0,21	0,14	0,20	0,53	1,00				
13A	0,10	0,11	0,10	0,20	0,20	0,20	0,11	0,12	1,00			
13B	0,08	0,09	0,09	0,18	0,17	0,17	0,16	0,20	0,71	1,00		
14A	0,11	0,09	0,12	0,18	0,14	0,18	0,11	0,11	0,21	0,17	1,00	
14B	0,09	0,10	0,09	0,14	0,11	0,14	0,08	0,10	0,24	0,19	0,81	1,00

CXS

Sample	15A	15B	15C	16B	16C	16D	17A	17B	18A	18B	19A	19B	20A	20B
15A	1,00													
15B	0,68	1,00												
15C	0,62	0,73	1,00											
16B	0,36	0,33	0,26	1,00										
16C	0,28	0,24	0,23	0,32	1,00									
16D	0,29	0,20	0,23	0,24	0,53	1,00								
17A	0,36	0,41	0,33	0,38	0,30	0,31	1,00							
17B	0,27	0,35	0,29	0,23	0,27	0,23	0,47	1,00						
18A	0,32	0,30	0,27	0,27	0,17	0,18	0,25	0,17	1,00					
18B	0,34	0,32	0,29	0,24	0,18	0,19	0,25	0,18	0,93	1,00				
19A	0,22	0,30	0,23	0,27	0,19	0,18	0,33	0,27	0,19	0,16	1,00			
19B	0,29	0,34	0,30	0,37	0,31	0,35	0,56	0,51	0,15	0,14	0,39	1,00		
20A	0,12	0,09	0,09	0,20	0,16	0,15	0,21	0,16	0,15	0,15	0,23	0,19	1,00	
20B	0,16	0,12	0,13	0,17	0,15	0,18	0,23	0,17	0,13	0,13	0,23	0,22	0,79	1,00
