# Supplementary material for: Different patterns of clonal evolution among different sarcoma subtypes followed for up to 25 years
Source: Nat Commun. 2018 Sep 10;9:3662. doi: 10.1038/s41467-018-06098-0 (PMC6131146; doi:10.1038/s41467-018-06098-0)
Supplement: Supplementary file 3 — Description of Additional Supplementary Files [file 41467_2018_6098_MOESM3_ESM.zip]

**Description of Additional Supplementary Files**

File Name: Supplementary Data 1

Description: SNP array intrasample heterogeneity.

File Name: Supplementary Data 2

Description: Genomic imbalances detected by SNP array analysis in multiple samples from sarcomas.

File Name: Supplementary Data 3

Description: Results of whole exome (WES) and targeted re-sequencing (TSCA).

File Name: Supplementary Data 4

Description: Clinical data and summary of genetic analyses performed.

File Name: Supplementary Data 5

Description: Minimally gained segments in chromosome 12 in 12 samples from welldifferentiated liposarcomas.
